# Beyond the Myths: Brazilian Consumer Perceptions of Functional Food

**DOI:** 10.3390/foods13244161

**Published:** 2024-12-22

**Authors:** Luis Gustavo Saboia Ponte, Suliene França Ribeiro, Adriane Elisabete Costa Antunes, Rosangela Maria Neves Bezerra, Diogo Thimoteo da Cunha

**Affiliations:** 1 Laboratório Multidisciplinar em Alimentos e Saúde, Faculdade de Ciências Aplicadas, Universidade Estadual de Campinas (UNICAMP), Limeira 13484-350, SP, Brazil; lg_saboia@hotmail.com (L.G.S.P.); sulienefr@gmail.com (S.F.R.); rbezerra@unicamp.br (R.M.N.B.); 2 Laboratório de Lácteos, Prebióticos e Probióticos, Faculdade de Ciências Aplicadas, Universidade Estadual de Campinas (UNICAMP), Limeira 13484-350, SP, Brazil; adriane@unicamp.br

**Keywords:** consumer perception, beliefs, consumer study, healthy eating, bioactive compounds

## Abstract

The growing consumer interest in functional foods and healthy eating can unfortunately lead to the spread of misinformation and the belief in food-related myths. This study analyzed Brazilian consumers’ perceptions and beliefs about facts and myths regarding functional foods, focusing on attitudes, reference groups, and sociocultural factors affecting their perception. A theoretical model was developed, incorporating constructs such as attitudes (reward, trust, necessity, safety), beliefs, and reference groups. Data from 600 participants in the Southeast (*n* = 300) and Northeast (*n* = 300) of Brazil were collected through online questionnaires, with responses measured on a five-point Likert scale. Myths (widely held ideas lacking scientific basis) and ‘facts’ (evidence-based information) regarding functional food were selected via literature review and validated by nutrition experts. Structural equation modeling revealed that perceived necessity and reward were positively associated with myths, while safety perception was negatively associated with myths. Reference groups and beliefs were positively associated with facts. Cluster analysis identified two consumer profiles: (1) safety-conscious individuals, who prioritize food safety, and (2) engaged critics, influenced by reference groups and actively seeking information. These findings highlight the importance of culturally tailored communication strategies for countering myths and promoting functional foods in Brazil. Regulatory bodies in Brazil must enhance oversight of health claims to build consumer trust and encourage informed choices, fostering mindful consumption habits.

## 1. Introduction

Functional foods emerged in Japan in the 1980s to meet public health demands triggered by an aging population and rising healthcare costs. In 1991, the Japanese government introduced the FOSHU (Foods for Specified Health Use) label to identify foods that not only provide essential nutrients but also have specific health benefits, such as preventing disease and supporting the immune system [1]. The Japanese model served as the basis for a global understanding of functional foods and emphasized the role of scientifically proven health benefits. This initiative has inspired other countries to develop their own regulations tailored to local needs and circumstances. In Brazil, the European Union, and the United States, for example, regulations began to require that health claims be supported by sound scientific evidence [2,3,4]. This global progress has solidified the concept of functional foods, promoted public health policy, and driven innovation in the food industry to promote wellness and disease prevention [5,6]. However, adapting these frameworks to local circumstances is crucial to ensure effective public understanding and acceptance in countries with different cultural and socioeconomic contexts, such as Brazil.

In this context, the Brazilian National Health Surveillance Agency (ANVISA in Portuguese) is responsible for regulating functional foods in Brazil. ANVISA establishes standards based on scientific evidence to ensure the safety and efficacy of products with functional claims. Rather than providing a comprehensive definition of functional foods, ANVISA categorizes claims into two types: functional claims and health claims. Functional claims refer to the role of a nutrient or component in normal bodily functions, while health claims indicate a relationship between food consumption and the prevention of disease or the promotion of health. This classification system provides consumers with clear indications of the specific benefits associated with functional foods [7,8]. While these categories provide regulatory clarity, they may not be fully aligned with consumer understanding due to their technical nature, which can lead to misunderstandings. Simplifying and contextualizing this information for the public could improve understanding and trust. These regulations aim to protect consumers and make it easier for them to make food choices by ensuring the reliability of functionality claims [9]. These regulations aim to protect consumers and facilitate food choices, ensuring the reliability of functionality claims. Many consumers still find it difficult to fully understand the concept of functional foods. They often rely on myths—widely held beliefs with no scientific basis—which distort their perception of these products and lead to misinterpretations [10]. The dissemination of inaccurate information not only makes it more difficult for consumers to access reliable information, but also reduces the acceptance of and compliance with functional foods [11]. These food myths are widespread in Brazil and reflect the cultural and socioeconomic diversity of a country with five major regions (North, Northeast, Center-West, Southeast, and South), where dietary habits vary according to traditions, cultural values, and economic conditions [12,13].

This diversity also affects the understanding and acceptance of functional foods [14]. In the Southeast, home to 41% of the Brazilian population (85 mi. inhabitants) and where the majority of the national Gross Domestic Product is generated, access to health campaigns and nutritional information tends to facilitate the acceptance of these foods [12,15]. In contrast, in the Northeast, which comprises 27% of the population (53 mi. inhabitants) and has a relatively lower Human Development Index (HDI), limited resources and traditional dietary practices may result in functional foods being less understood or viewed with skepticism [12,15,16]. In this sense, in the Northeast, traditional food customs and a lower HDI often hinder the acceptance of functional foods, while in the Southeast, better access to health campaigns facilitates understanding and acceptance. These regional contrasts illustrate the complexity of the functional foods market in Brazil and underscore the importance of considering cultural and regional factors in understanding consumer perceptions. These differences highlight the uneven distribution of public knowledge about functional foods in Brazil and emphasize the need for educational initiatives that include culturally relevant messages. By addressing the specific regional challenges, such initiatives could help to reduce misunderstandings and promote informed choices [17]. This complexity also reflects the broader cultural influences that shape consumer perceptions and highlights the need for a regionally tailored approach [18,19].

Brazilian cuisine is a vibrant mix of indigenous, African, and European influences, resulting in a diverse culinary landscape [20,21]. In addition, many plants native to Brazil, such as “açaí” and “guaraná”, are used in traditional medicine and modern dietary supplements. However, due to popular belief, some foods are said to have false or unproven health-promoting properties, e.g., that peanuts or shellfish have an aphrodisiac effect or that coconut oil can help with weight loss. This cultural diversity, while enriching the national food identity, also contributes to misconceptions about functional foods, as traditional beliefs sometimes clash with scientific evidence. Thus, the diversity of perceptions among Brazilian consumers is influenced by cultural factors, pervasive myths, and socioeconomic barriers [22]. Many Brazilians attribute health benefits to popular and traditional foods, believing these offer protective functions, though this perception is not always scientifically supported and often overlooks the additional properties of foods regulated as functional [23]. This may even occur with health professionals [24]. Furthermore, the dissemination of misinformation hinders the comprehension of functional concepts, thereby limiting the adoption and utilization of these products [25]. When professionals are inadequately informed, they may inadvertently perpetuate these myths, underscoring the need for continuous professional development and evidence-based education. By using validated myths and facts, this study differentiates cultural beliefs from evidence-based findings. The inclusion of peer-reviewed myths and region-specific data highlights how cultural narratives and traditions influence misconceptions and allows for targeted strategies to bridge gaps between traditional knowledge and scientific evidence. In addition, we seek to examine how attitudes, reference group influence, beliefs, and sociocultural factors impact the acceptability and consumption of these foods. The aim is to identify strategies that promote evidence-based information and reduce the spread of myths.

In order to achieve these aims, the study begins with a review of the relevant literature, which theoretically underpins and justifies the proposed hypotheses. The methodology is then described, detailing the process of data collection and the analytical techniques used. The results section presents the main findings, including the findings from structural equation modeling and cluster analysis. In the discussion, these results are interpreted, and implications and limitations are highlighted. Finally, the conclusion summarizes the main contributions of the study and makes suggestions for future research and policy development.

## 2. Literature Review and Research Hypotheses

Consumer perception of functional foods is a rapidly growing area, reflecting the increasing global interest in healthier eating and disease prevention [26]. Functional foods have attracted considerable attention as consumers strive to improve their quality of life and longevity [27]. However, accessibility and consumption of these foods remain unevenly distributed and are influenced by a complex interplay of factors such as individual attitudes, demographic characteristics, and sociocultural influences [28,29].

Current research shows that a positive perception of functional foods is not only related to knowledge, but also to consumers’ beliefs and attitudes, as well as the influence of reference groups on consumption decisions [30,31]. To better understand how cultural factors, consumer beliefs, and social influences shape the perception of functional foods, this study proposes a model that examines consumers’ attitudes toward functional foods, their beliefs about their benefits, the role of affinity groups in their food choices, and perceptions of food myths and facts. It is noteworthy that in this study, myths are defined as widely held beliefs with no scientific basis that distort understanding and lead to misinterpretation [32], e.g., that gluten-free products are better for health than foods with gluten. In contrast, facts are defined as scientifically proven claims that demonstrate certain health benefits, e.g., that fiber intake is important for normal bowel function.

Consumer attitudes play a significant role in the choice of functional foods. Attitudes, which reflect individuals’ predispositions to evaluate specific objects or concepts positively or negatively, are shaped by a mix of factors such as perceived health benefits, convenience, and the sensory characteristics of functional foods [33,34]. The Theory of Planned Behavior posits that attitudes, deeply influenced by an individual’s knowledge and beliefs, are paramount in shaping behavioral intentions and subsequent decisions. This theoretical framework strongly supports the idea that positive perceptions of functional foods arise from the congruence between consumers’ attitudes and scientifically substantiated claims. Moreover, the theory underscores the significant impact of subjective norms, encompassing the influence of reference groups, on decision-making processes. This highlights the crucial role of both factual information and circulating myths about functional foods within these social contexts [35]. Urala and Lahteenmäki (2007) [36] identify four main factors that shape consumer attitudes toward functional foods: perceived reward from consumption, the perceived necessity of including these foods in the diet, trust in claimed benefits, and the level of perceived safety. In this context, the perception of reward from consuming functional foods, based on scientifically proven benefits, is expected to foster positive attitudes and a greater intention to choose these foods. Safraid et al. (2024) [37] and Dolgopolova et al. (2015) [38] also emphasize that trust and a sense of safety act as cognitive filters that enable consumers to critically evaluate claims and distinguish credible information from misleading or exaggerated claims. This ability to evaluate reliability not only promotes evidence-based consumption, but also strengthens consumer confidence in functional food choices. Based on this, we have our first hypothesis:

**Hypothesis** **1 (H1):** Attitudes—rewards (H1a), needs (H1b), trust (H1c), and safety (H1d)—toward functional foods predict positive perceptions regarding facts.

Conversely, the perceived need to include functional foods in the diet, without scientific validation of specific benefits for all consumers, may lead to the spread of unverified information about these foods (H2). This hypothesis is based on studies that emphasize the vulnerability of consumers in the search for health-related solutions. For example, Annunziata, Vecchio, and Kraus (2015) [39] found that perceived necessity often correlates with increased susceptibility to accepting untested claims, as individuals prioritize perceived benefits over scientific rigor. Thus, our second hypothesis is as follows:

**Hypothesis** **2 (H2):** Attitudes—rewards (H2a), needs (H2b), trust (H2c), and safety (H2d)—toward functional foods predict positive perceptions regarding myths.

Another factor influencing the choice of functional foods is the role of reference groups, including family, friends, and other influential figures in consumers’ lives [40]. These groups play a fundamental role in shaping food preferences and purchasing decisions, especially for foods perceived as health-beneficial. Social acceptance and the promotion of functional foods within a social circle can significantly increase an individual’s likelihood of choosing these products [41,42]. Consequently, this study hypothesizes that reference groups, when promoting unverified or exaggerated information about functional foods, influence the adoption of these products based on popular conceptions not always scientifically supported. Bandura’s (1971) [43] social learning theory supports this hypothesis by highlighting how behaviors and attitudes are shaped by social contexts. In addition, Wang et al. (2021) [44] provide empirical evidence that reference groups can reinforce both correct and myth-driven perceptions through social reinforcement mechanisms. Thus, our third hypothesis is as follows:

**Hypothesis** **3 (H3):** Reference groups predict positive perceptions regarding both facts (H3a) and myths (H3b).

Beliefs are understood as convictions about the truth or falsehood of a concept, and they can directly influence how consumers perceive and evaluate functional foods, thereby affecting acceptance and consumption [31]. Neupane, Chimhundu, and Chan [45] demonstrate that beliefs about health benefits, disease prevention, and trust in product claims are fundamental for the acceptance of these foods. Thus, consumer beliefs regarding health benefits and disease prevention, when supported by scientific research, directly impact the acceptance of functional foods. Verbeke (2005) [31] highlights that a lack of exposure to clear, evidence-based information often impairs consumers’ ability to differentiate facts from myths. This underscores the importance of beliefs supported by scientific research in fostering accurate perceptions, providing a strong foundation for Hypothesis 4.

**Hypothesis** **4 (H4):** Beliefs predict positive perceptions regarding facts (H4a) but not myths (H4b).

**Figure 1:**
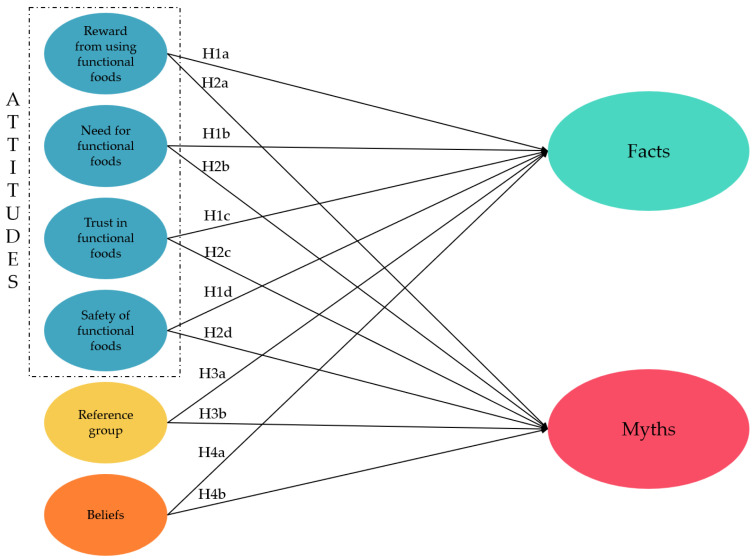
Proposed model.

These interconnected factors—attitudes, beliefs, and reference groups—are essential to understanding how consumers perceive and consume functional foods. Understanding these dynamics is crucial for developing strategies that promote the acceptance and consumption of these products, contributing to both public health outcomes and the growth of the functional foods market. For this investigation, the model shown in Figure 1 was proposed.

## 3. Methods

### 3.1. Sample and Data Collection

The data were collected via the online platform Google Forms (Alphabet Inc., Mountain View, CA, USA). Initially, a pilot test was conducted with twenty people to assess the clarity of the questionnaire and the average response time (≈15 min). Sampling was purposive, non-probabilistic, and performed using a chain referral method. Invitations were sent via Facebook, Instagram, WhatsApp, and SMS. The inverse square root method [46] was used to determine a minimum sample size of 155 participants per group, based on a significance level of 5% and a path coefficient of 0.20. A larger sample size was used to account for the potential variability in the non-probabilistic sample.

Without restrictions on gender or educational level, the survey collected 600 questionnaires between February and October 2023, with the sample evenly distributed between the Southeast (*n* = 300) and Northeast (*n* = 300) regions of Brazil. To avoid monotonous answers, some questions contained reverse-scored items. In addition, the standard deviation (SD) of the indicator variables was checked for each participant. No participant had an SD = 0, indicating a monotonic response. All participants signed a consent form electronically. The study was approved by the Ethics Committee of the University of Campinas (protocol: 58614722.6.0000.5404; 10 January 2023).

### 3.2. Measures

To test the research hypotheses, a 62-question questionnaire (Table S1) was completed, divided into two parts. The first part included 8 questions on sociodemographic characteristics (e.g., gender, age, level of education, professional background, etc.). The second part consisted of 54 measurement items as indicator variables taken from studies. This part included constructs for attitude (28 indicators; Urala and Lähteenmäki [36]), reference group (6 indicators; Salmani et al. [47]), and beliefs (4 indicators; Verbeke, 2005 [31]).

The myths and facts were selected on the basis of a thorough review of the literature in scientific databases, social networks, and popular newspapers. A list of 13 myths and 3 facts was then reviewed and validated by a group of four nutrition experts. This ensured that the included myths reflect popular beliefs that have no scientific basis, while the facts are based on scientific evidence.

All indicators were rated on a five-point Likert scale, ranging from 1 = strongly disagree to 5 = strongly agree.

### 3.3. Data Analysis

First, a confirmatory factor analysis (CFA) was conducted using the diagonally weighted least squares (DWLS) method to assess the factors in the model. This analysis was necessary because the questionnaire was adapted for the Brazilian sample, so it was essential to verify the suitability of its factor structure. It was expected that each indicator would have a factor loading of over 0.50. The fit of the model was assessed using the following parameters: chi-square (χ^2^, *p* < 0.05), comparative fit index (CFI > 0.95), root mean square error of approximation (RMSEA < 0.08), Tucker–Lewis index (TLI > 0.90), standardized root mean square residual (SRMR < 0.08), and goodness of fit index (GFI > 0.95), as recommended by Byrne [48].

To test the hypotheses of the theoretical model, a partial least squares structural equation modeling (PLS-SEM) approach was applied. This method offers greater flexibility, especially for small samples and cases where variables do not follow a normal distribution [49]. The first prerequisite for PLS-SEM is a clear theoretical model that describes the relationships between latent variables. Second, while PLS-SEM is often less stringent than covariance-based SEM in terms of sample size, a general guideline is that the number of indicators for the construct with the most indicators should be at least 10 times as large. PLS-SEM can handle different types of data scales and does not necessarily require normally distributed data. Finally, PLS-SEM assumes linear relationships between the constructs [49]. The measurement model, which defines the relationship between latent variables and their respective indicators, was evaluated based on factor loadings (>0.60), composite reliability (CR > 0.70), and average variance extracted (AVE > 0.50), following Fornell and Larcker’s [50] recommendations. Discriminant validity was checked using the heterotrait–monotrait ratio (HTMT), with values below 0.85 [49,51].

The structural model, demonstrating relationships between latent variables, was analyzed based on the proportion of explained variance in endogenous constructs, effect size (f^2^ > 0.15), and predictive relevance (Stone–Geisser Q^2^ > 0). Effect size was classified according to Cohen [52], as small (f^2^ ≥ 0.02), medium (f^2^ ≥ 0.15), or large (f^2^ ≥ 0.35). The significance of paths was evaluated using bootstrapping with 5000 resamples, with the significance level set at *p* < 0.05. All statistical analyses were performed using SmartPLS v4 software (SmartPLS GmbH, Bönningstedt, Germany), as per Ringle et al. [53].

Finally, a segmentation of participants was performed using a cluster analysis with the k-means clustering method, which aims to divide the dataset into k clusters so that the sum of squared distances between each point and its cluster center is minimized [54]. To ensure comparability of values and to account for possible differences in scale between variables, the data were standardized using Z-scores. The optimal number of clusters was determined using the silhouette coefficient, which evaluates the cohesion and separation of clusters. The silhouette index ranges from −1 to 1, with values close to 1 indicating high cohesion within clusters and good separation between clusters [55]. Once the groups were formed, contingency tables were created showing the frequency of participants in each cluster, categorized by subgroups of the sample. Statistical analyses were performed using JASP 0.18.2 software from the University of Amsterdam.

## 4. Results

### 4.1. Sample Characterization

The sample consisted of 600 individuals, the majority of whom were female (57.8%) and single (62.8%). Regarding age, 41.8% were young adults (18–29 years), 55.8% were adults (30–59 years), and 2.3% were elderly (≥60 years). The sample was considered highly educated, with 69.2% holding a college degree or postgraduate qualification (Table 1).

### 4.2. Confirmatory Factor Analysis

Table 2 presents the indicators and respective constructs evaluated. The model showed an adequate fit in the CFA with: χ^2^ = 2554.56 (*p* < 0.001), CFI = 0.970, TLI = 0.968, RMSEA = 0.061, SRMR = 0.022, and GFI = 0.973. The model was confirmed, and subsequently, the factors were included in the PLS-SEM for the measurement model.

### 4.3. Measurement Model

The constructs showed satisfactory reliability levels, with CR values above 0.7 (Table 3). The AVEs of factors 1 to 4 exceeded the value of 0.50, indicating adequate convergent validity. The AVEs for factors 5 to 7 were suboptimal and exceeded the value of 0.45. According to Fornell and Larcker (1981) [50], the convergent validity of the construct is still sufficient if the AVE is below 0.5 but the CR is above 0.6. Additionally, HTMT values ranged from 0.098 to 0.696, remaining below the 0.85 threshold, confirming adequate discriminant validity (Table 3).

### 4.4. Structural Model

**Figure 2:**
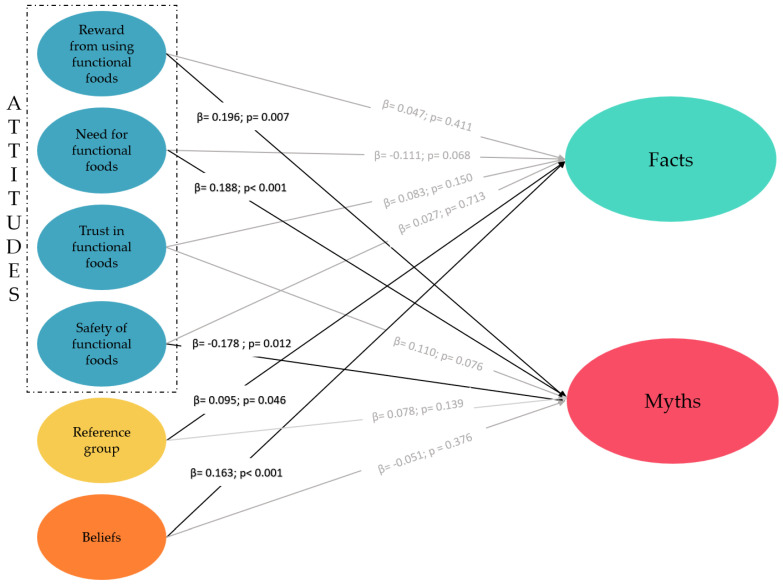
Final structural model.

In the structural model (Figure 2), significant paths were identified among the evaluated constructs, highlighting key relationships in the perception of functional foods. The model showed adequate predictive relevance (Q^2^ = 0.11). The hypotheses H2a, H2b, H2d, H3a, and H4a were confirmed. Consumer attitudes toward functional foods, specifically the need for, reward derived from their use, and safety, were differentially associated with myth perceptions: need (β = 0.188; *p* < 0.001; f^2^ = 0.03) and reward (β = 0.196; *p* = 0.007; f^2^ = 0.02) were positively correlated, while safety was negatively correlated (β = −0.178; *p* = 0.012; f^2^ = 0.01). Additionally, both the reference group (β = 0.09; *p* = 0.04; f^2^ = 0.01) and beliefs about functional foods (β = 0.163; *p* < 0.001; f^2^ = 0.01) demonstrated positive associations with facts, indicating that individuals influenced by their social networks or who have a strong interest in these foods are more aligned with evidence-based information. While not significant, a tendency was observed with a negative correlation between need and facts (β = −0.111; *p* = 0.068; f^2^ = 0.01), and a positive correlation between safety and facts (β = 0.110; *p* = 0.07; f^2^ = 0.01). 

It is important to note that most of the significant effects had small effect sizes, suggesting that while the independent factors have a statistically significant relationship with the perception of myths and facts, the practical significance of these relationships is limited. Both models showed a modest determination coefficient with R^2^ = 0.09 for myths and R^2^ = 0.14 for facts.

### 4.5. Cluster Analysis

Cluster analysis was performed using centered scores (M = 0, SD = 1) of studied factors. The silhouette method was used to determine the optimal number of clusters, indicating the formation of two clusters.

Based on this, participants were segmented into two clusters using the K-means method. Table 4 presents the mean scores for each dimension by cluster. Cluster 1, labeled as “safety-cautious”, includes individuals with low scores in rewards and trust about functional foods, reference group, and beliefs but with positive scores in need and safety of functional foods. Cluster 2, labeled as “engaged and critical”, comprises individuals with positive scores in reward, trust, and reference group but with negative scores in need and safety of functional foods. Both clusters had mean scores close to zero for the myths variable.

The clusters are presented in a two-dimensional space, where the principal components explain 35.6% and 23.7% of the data variance, respectively. Despite the segmentation, there is a significant overlap between the two clusters, suggesting limited differentiation between them and indicating that the distinction between groups may not be substantial.

Table 5 shows the distribution of individuals by cluster according to sample subgroups. Both clusters are well represented across most subgroups, with the engaged and critical cluster having a larger number of individuals (*n* = 371) compared to the safety-cautious group (*n* = 229). Additionally, some differences in cluster composition were observed: Cluster 2 has a higher proportion of individuals from the Northeast region (χ^2^ = 4.41; *p* = 0.036) and Cluster 1 has younger people (t = 2.43; *p* = 0.015).

## 5. Discussion

### 5.1. General Discussion

The analysis of consumer perceptions and beliefs reveals that varied motivations influence the interpretation of information about functional foods. The structural model presented highlights significant connections among the constructs of attitude (need and reward), reference group, and beliefs, demonstrating how these factors shape interpretations of myths and facts associated with functional foods. Although the results were statistically significant, they indicate a relatively small effect size. This suggests that while attitudes, reference group influence, and beliefs are important, they are unlikely to be the only determinants of perceptions of myths and facts about functional foods. Understanding these relationships is essential for developing more effective communication and educational strategies that foster evidence-based perceptions and reduce the spread of misinformation about food functionality [56,57]. However, further research is needed to identify additional factors that may play a greater role.

The need factor showed a positive association with myths, indicating that personal perception of need can lead some consumers to accept unverified information [58,59]. This behavior may be related to the pursuit of quick solutions for health issues, making these individuals more susceptible to popular ideas and unproven promises [60]. This highlights the role of culturally rooted beliefs in shaping perceptions, as many Brazilian consumers attribute functional properties to traditional foods without any scientific basis. For example, foods such as *guarana* and *açaí* are widely believed to have health benefits, although there is little evidence to support these claims. In this context, Çakiroğlu and Uçar [61] highlight that many consumers view functional foods as essential for a healthy diet, driven by the desire for health and prosperity—an idea supported by studies by Landström et al. [62], Karelakis et al. [63], and Plasek et al. [64].

In this regard, the reward factor also showed a positive association with myths, indicating that consumers who view functional foods as a form of self-care and well-being may be more inclined to accept scientifically unsupported information. This behavior is observed in studies by Bekoglu et al. [65] and Carrillo et al. [66], which identified the perception of reward as an important predictor for functional food consumption, driven by benefits such as enhanced performance, improved mood, and disease prevention. Thus, the perception of reward can foster a positive relationship with these foods, though it also suggests the need for more informed guidance to promote conscious consumption [67]. In contrast, the negative association between the safety and myths factors suggests that consumers with a heightened concern for safety tend to be more critical of unverified information about functional foods. This critical view is more prevalent in the southeastern region, where access to health education is better, than in the Northeast, where traditional customs and limited health knowledge can lead to misinformation. These consumers, by prioritizing safety and quality assurances, demonstrate a lower tendency to accept unsubstantiated claims or exaggerated promises. This stance aligns with findings from studies by Gajaria and Mantri [68] and Chen and Martirosyan [28], which show that safety-conscious consumers seek greater scientific backing and reliability in information about functional foods. In this way, the perception of safety acts as a filter, helping these individuals better discern between facts and myths, fostering more discerning and evidence-based consumption [69]. This underscores the importance of clear and substantiated communication about functional foods, aligning consumer expectations with scientific evidence.

The influence of the reference group on facts suggests that the social environment plays an important role in disseminating reliable information about functional foods. Individuals who are part of networks of informed people tend to have scientifically grounded perceptions [38]. This influence is supported by studies such as those of Febian et al. [70] and Nguyen et al. [71], which highlight the impact of social norms on food choices, and that of Barauskaite et al. [72], which reinforces the role of family and friends in shaping food attitudes.

Additionally, beliefs about functional foods showed an association with facts, indicating that consumers with a greater interest in this type of food tend to distinguish between evidence-based information and misconceptions [59]. Studies like that of Topolska et al. [73] indicate that, although consumers trust the benefits of functional foods, they are often influenced by marketing campaigns that exaggerate their effects. This scenario leads to a distorted understanding, creating unrealistic expectations and making consumers vulnerable to unproven promises. To strengthen trust, it is essential that campaigns are based on scientific evidence and promote clear and realistic information, helping the public make informed choices.

Finally, the segmentation of participants into two distinct clusters using the K-means method revealed two well-defined profiles in perceptions of functional foods. The first group, labeled safety-conscious, includes individuals who scored low in dimensions of beliefs, need, and reference group, but had high scores in safety. This profile suggests a more conservative stance, where, despite lower confidence in the beliefs and need for functional foods, safety is highly valued as a central criterion in food choices. Public campaigns targeting this group should emphasize the safety and scientifically proven benefits of functional foods and build trust through clear and transparent communication. When functional foods are perceived as safe, consumers tend to trust their potential health benefits, viewing these advantages as a form of reward [74]. This cluster presents an opportunity for educational interventions that could strengthen positive beliefs about the functionality of these foods, provided that safety and reliability remain emphasized.

In contrast, the second group, labeled engaged and critical, comprises individuals with high involvement in functional foods, as indicated by positive scores in beliefs, need, and reference group. However, these participants scored lower in safety, suggesting that, despite their interest and perceived need, doubts remain about the potential risks of these foods. This critical stance aligns with studies by Barreiro-Hurlé et al. [75], Huang et al. [76], and Kavoosi-Kalashami et al. [77], which observed that highly engaged functional food consumers often take a cautious view on safety due to perceived risks. For this group, more transparent communication strategies that emphasize safety and real benefits could be effective, fostering greater trust and acceptance. The segmentation into safety-conscious and engaged and critical offers valuable insights for designing targeted marketing and nutritional education strategies. Understanding these differences enables the development of tailored approaches that can address both safety concerns and genuine interest in functional foods.

The demographic analysis also revealed trends linked to cultural and sociodemographic factors. For example, the greater presence of women in Cluster 2 aligns with research indicating that women tend to show more interest in food and health issues compared to men [78]. The absence of individuals with children or dependents in this group suggests a profile focused more on self-care and individualized dietary choices. Additionally, the predominance of consumers from the Northeast in Cluster 2 reflects the growing demand for functional foods in the region, similar to trends observed in other emerging economies [59,79]. These sociodemographic differences—encompassing age, gender, income, and education level—are fundamental to guiding the development of product offerings and effective communication strategies to encourage greater acceptance and utilization of this food category.

### 5.2. Practical and Policy Implications

The results of this study indicate that communication and public health education strategies should be regionalized and tailored to cultural specificities to promote informed consumption of functional foods in Brazil. The finding that reference groups and sociocultural factors influence the trust and acceptability of these foods suggests that educational campaigns promoting functional foods may be more effective when aligned with the local values and practices of each region. Therefore, it is recommended that public health authorities and agencies collaborate with community leaders and local influencers to convey information about the proven benefits of these foods, countering myths and misinformation that hinder understanding and adherence to the concept of food functionality.

At the policy level, the data on differences in acceptability and knowledge between regions, such as the Southeast and Northeast, reinforce the need for public policies that prioritize equal access to information and healthy foods. To address regional inequalities, it is recommended that government initiatives and partnerships with producers work to promote economic access and availability of functional foods in regions with lower economic development. Such policies could include tax incentives for companies offering products with functional properties, as well as the creation of subsidy programs to increase access to health-promoting foods, especially for socially vulnerable populations.

Finally, from a regulatory standpoint, the findings on the persistence of myths and misconceptions suggest the importance of strengthening oversight of health claims on functional foods. It is essential that ANVISA and other responsible agencies reinforce monitoring of advertising and health information for this group of foods, ensuring that claims are always supported by robust scientific evidence. Additionally, educational actions that guide consumers toward reliable information could help reduce the spread of myths and strengthen public trust in functional foods, contributing to more informed and healthier food choices.

### 5.3. Limitations and Further Research

The first limitation relates to the effect size of the model paths. Although significant relationships were identified, some individual paths had weak effect sizes (f^2^ < 0.02). In addition, the overall model fit, as indicated by the coefficient of determination (R^2^), was modest, explaining only 9% and 14% of the variance in myth and fact perceptions, respectively. These results emphasize the need to investigate additional, and thus stronger, factors and variables that might influence these perceptions.

The study was only conducted in two regions of Brazil using non-probabilistic samples, which limits the generalizability of the results. Despite this limitation, the results provide valuable insights into consumer perceptions. Future studies that expand the geographic diversity of the sample and enable international comparisons could contribute to a broader understanding of consumer motivations and preferences, supporting the development of educational and market strategies better aligned with each consumer profile’s specific needs.

## 6. Conclusions

This study examined Brazilian consumers’ perceptions of functional foods, considering social and regional influences. The analysis showed that factors such as reference groups and trust in health information differ between the Southeast and Northeast regions, highlighting contextual nuances in the consumption of these foods. Consumer segmentation revealed two distinct profiles: “safety-cautious” and “engaged and critical”, indicating that expectations and receptiveness toward functional foods are shaped by beliefs and safety concerns. Perceptions of health and wellness benefits were found to be relevant to the acceptance of these foods, although myths and misinformation still impact consumption. These findings suggest that communication strategies tailored to regional and cultural contexts could foster a more accurate understanding of food functionality. To deepen this area, future research should explore other regions of the country and consider international comparisons, providing a broader foundation for public policy and educational initiatives that encourage informed consumption of functional foods.

## Figures and Tables

**Table 1:** Sample characteristics.

Variable	*n*	%	Variable	*n*	%
**Gender**			**Education Level**		
Male	250	41.8	Incomplete elementary school	2	0.3
Female	345	57.8	Complete elementary school	5	0.8
Non-binary	1	0.2	Incomplete high school	6	1
Prefer not to say	4	0.7	Complete high school	57	9.5
**Marital Status**			Incomplete higher education	115	19.2
Single	377	62.8	Complete higher education	160	26.7
Married	133	22.5	Postgraduate	255	42.5
Divorced	24	4	**Monthly Household Income**		
Widowed	6	1	No income	2	0.3
Common-law marriage	60	10	Up to 1 minimum wage (MW)	27	4.5
**Age**			From 1 to 3 MW	83	13.8
18–29 years	251	41.8	From 3 to 5 MW	120	20
30–39 years	252	42	From 5 to 7 MW	120	20
40–49 years	59	9.8	From 7 to 10 MW	113	18.8
50–59 years	54	9	More than 10 MW	135	22.5
60 years or older	14	2.3	**Are there children and/or elderly people living in your household?**		
**Worked in the food sector?**			No	363	60.5
No	457	76.2	Yes, children and elderly	35	5.8
Yes	143	23.8	Yes, children	101	16.8
			Yes, elderly	101	16.8

**Table 2:** Factor loadings, means, standard deviation, composite reliability, and average variance extracted for constructs and indicators.

Factor	Indicators	Factor Loading	Mean ± SD
**Attitude**	**Reward from using functional foods**		
	Functional foods help improve my mood.	0.761	4.185 ± 0.938
	My performance improves when I consume a functional food.	0.773	4.360 ± 0.932
	Functional foods facilitate the adoption of a healthy lifestyle.	0.784	4.555 ± 0.865
	I can prevent diseases by regularly eating functional foods.	0.790	4.512 ± 0.915
	I enjoy the idea that I can take care of my health by eating functional foods.	0.723	4.322 ± 1.021
	Functional foods can repair damage caused by an unhealthy diet.	0.594	3.887 ± 1.180
	Even if the taste is bad, I will consume a food with functional characteristics.	0.528	3.068 ± 1.332
	I actively seek information about functional foods.	0.695	3.137 ± 1.382
	**Need for functional foods**		
	Functional foods are completely unnecessary. *	0.904	4.615 ± 0.928
	Functional foods are a complete sham. *	0.918	4.698 ± 0.793
	The increasing number of functional foods on the market is a bad trend for the future. *	0.691	4.248 ± 1.226
	It is not appropriate to associate foods with health effects. *	0.513	4.420 ± 1.072
	Functional foods are mainly consumed by people who don’t need them. *	0.552	3.878 ± 1.167
	**Trust in functional foods**		
	Functional foods promote my well-being.	0.842	4.385 ± 0.821
	The information available on the effects of functional foods is reliable.	0.671	3.700 ± 0.992
	The use of functional foods is completely safe.	0.760	3.895 ± 1.021
	The safety of functional foods has been well studied.	0.678	4.013 ± 0.963
	I believe that functional foods fulfill their promised functions/characteristics.	0.843	4.057 ± 0.861
	Functional foods are primarily science-based products.	0.600	3.423 ± 1.060
	**Safety of functional foods**		
	If overused, functional foods can be harmful to health.	0.634	2.727 ± 1.337
	In some cases, functional foods can be harmful to healthy people.	0.829	3.265 ± 1.351
	The consumption of functional foods is completely safe. *	0.871	3.450 ± 1.107
	The new functionalities analyzed in foods bring unforeseen risks.	0.472	3.013 ± 1.320
**Reference group**	My family’s view is that eating functional foods is beneficial to health.	0.788	4.008 ± 1.112
	Today, most health professionals approve of consuming functional foods.	0.785	4.035 ± 0.977
	My friends eat functional foods.	0.529	3.388 ± 1.109
	My family encourages me to consume functional foods.	0.801	3.392 ± 1.321
	Most people who care about me think that eating functional foods is good for health.	0.836	3.722 ± 1.206
	Nutritionists recommend consuming functional foods.	0.869	4.243 ± 0.930
**Beliefs**	Functional foods will likely have a beneficial impact on my health.	0.881	4.512 ± 0.786
	I try functional foods in an attempt to pursue a healthy lifestyle.	0.832	4.210 ± 1.032
	Consuming functional foods allows me to take care of my health.	0.855	4.185 ± 0.976
	Including functional foods in my routine helps me meet my daily needs.	0.869	4.173 ± 0.953
**Myths**	Gluten makes you gain weight and is harmful. *	0.590	2.490 ± 1.366
	The consumption of milk and dairy products causes inflammation in the body. *	0.504	2.737 ± 1.370
	Coconut oil aids in weight loss.	0.744	2.338 ± 1.297
	Eggplant water helps with weight loss and reduces cholesterol.	0.843	2.678 ± 1.264
	Chicken meat is harmful due to the addition of hormones in these animals. *	0.481	2.095 ± 1.206
	Gelatin helps increase skin elasticity due to collagen.	0.645	2.608 ± 1.431
**Facts**	Carrots are good for vision and skin.	0.680	4.098 ± 1.062
	Garlic helps reduce blood pressure and lowers LDL (bad cholesterol) while increasing HDL (good cholesterol).	0.604	3.585 ± 1.107
	Fiber intake is important for normal bowel function.	0.690	4.698 ± 0.801

* reversed indicators.

**Table 3:** Composite reliability (CR), average variance extracted (AVE), and discriminant validity with heterotrait–monotrait ratio (HTMT) of correlations.

Factors	CR	AVE	HTMT of Correlations						
			(1)	(2)	(3)	(4)	(5)	(6)	(7)
Trust in functional foods (1)	0.878	0.546	1.000						
Beliefs (2)	0.908	0.712	0.689	1.000					
Facts (3)	0.780	0.543	0.381	0.448	1.000				
Reference group (4)	0.880	0.553	0.578	0.659	0.357	1.000			
Need for functional foods (5)	0.820	0.480	0.248	0.379	0.262	0.251	1.000		
Reward from using functional foods (6)	0.823	0.488	0.696	0.618	0.352	0.470	0.311	1.000	
Safety of functional foods (7)	0.778	0.480	0.278	0.186	0.111	0.201	0.305	0.134	1.000
Myths (8)	0.829	0.453	0.159	0.098	0.351	0.142	0.157	0.194	0.149

**Table 4:** Mean scores (Z-score) by cluster for each dimension in the food functionality perception questionnaire.

Factors	Cluster 1 (*n* = 229)	Cluster 2 (*n* = 371)
Reward	−0.69	0.43
Need	0.31	−0.19
Trust	−0.83	0.51
Safety	0.32	−0.20
Reference group	−0.76	0.47
Beliefs	−0.85	0.53

**Table 5:** Percent distribution and counts by cluster for sample subgroups.

Subgroups	Overall Sample (%)	Cluster 1—Safety-Cautious (%)	Cluster 2—Engaged and Critical (%)
*Gender*			
Male	41.9	45.7	39.3
Female	58.1	54.3	60.7
*Region*			
Northeast	50.3	46.5	55.5
Southeast	49.7	44.5	53.5
*Education*			
Up to complete high school	11.5	10.0	12.5
Incomplete or complete higher education	45.8	49.8	43.0
Postgraduate	42.7	40.2	44.5
*Income*			
Up to 1 minimum wage (MW)	6.8	7.5	6.3
From 1 to 3 MW	27.5	24.5	29.6
From 3 to 5 MW	24.3	24.9	23.9
From 5 to 7 MW	14.7	14.1	15.1
From 7 to 10 MW	13.2	12.4	13.7
More than 10 MW	13.5	16.6	11.4
*Children and elderly at home*			
None	60.0	58.9	60.7
Children	17.1	16.2	17.7
Elderly	17.1	19.5	15. 4
Children and elderly	5.9	5.4	6.3
*Marital Status*			
Single	62.3	68.5	58.1
Married	32.6	28.2	5.6
Divorced	4.0	2,9	4.8
Widowed	1.0	0,4	1.4
*Age*			
18 to 29 years	41.7	44.9	9.6
30 to 39 years	41.9	42.0	41.9
40 to 49 years	10.0	9.5	10.3
50 to 59 years	4.0	1.7	20.0
60 years or older	2.4	2.1	5.7

## Data Availability

The original contributions presented in the study are included in the article, further inquiries can be directed to the corresponding author.

## Supplementary Materials

The following supporting information can be downloaded at: https://www.mdpi.com/article/10.3390/foods13244161/s1, Table S1: Questionnaire on Perceptions and Beliefs About Food Functionality, provides the complete questionnaire used in the study, covering topics such as socio-demographic characteristics, attitudes, reference groups, beliefs, facts, and myths.
