# Supplementary material for: Beyond the Myths: Brazilian Consumer Perceptions of Functional Food
Source: Foods. 2024 Dec 22;13(24):4161. doi: 10.3390/foods13244161 (PMC11675342; doi:10.3390/foods13244161)
Supplement: Supplementary file 1 [file foods-13-04161-s001.zip › foods-3381998-supplementary.pdf]

Table S1 - Questionnaire on Perceptions and Beliefs About Food Functionality

UNICAMP   CNEM Ciências da Nutrição e do Esporte e Metabolismo   LabMAS

You are invited to participate in a research study on Perceptions and Beliefs about Food Functionality. Your participation is very important to contribute to the objective of the study. The research focuses on your opinion, so there are no right or wrong answers. Please respond according to what you believe, providing the first answer that comes to mind. The estimated time required to complete the questionnaire is approximately 15 minutes.

Note: The research is anonymous and only individuals aged 18 or older who reside in the Northeast and Southeast regions are eligible to participate.

Are you 18 years old or older?

[YES]   [NO]

Do you consume foods that provide health benefits?

[YES]   [NO]

Which region do you live in?

[Southeast]   [Northeast]

Which city? Which state?

____

What is your email address?

____

What is your phone number?

____

1) Gender:

☐ Female;

☐ Male;

☐ Non-binary;

☐ Prefer not to say;

☐ Prefer to self-describe: ________________________

2) Age:

____

3) Education Level:

☐ Incomplete primary education;

☐ Complete primary education;

☐ Incomplete high school;

☐ Complete high school;

☐ Incomplete higher education;

☐ Complete higher education;

☐ Incomplete postgraduate education. Specify: ______________ (specialization/master’s/PhD);

☐ Complete postgraduate education. Specify: ______________ (specialization/master’s/PhD);

4) Monthly Household Income:

☐ Up to 1 minimum wage;

☐ 1 to 3 minimum wages;

☐ 3 to 5 minimum wages;

☐ 5 to 7 minimum wages;

☐ 7 to 10 minimum wages;

- [ ] More than 10 minimum wages;

5) Marital Status:

- [ ] Single
- [ ] Married
- [ ] Divorced
- [ ] In a domestic partnership
- [ ] Widowed
- [ ] Other: _______________.

6) Do children and/or elderly people live in your household?

- [ ] Yes, children;
- [ ] Yes, elderly people;
- [ ] Yes, both children and elderly people;
- [ ] No.

7) Occupation::

____

8) Have you ever worked in the food sector?

- [ ] Yes;
- [ ] No.

If yes, which of the following:

- [ ] Food industry;
- [ ] Food retail (supermarkets);
- [ ] Food service (restaurants, snack bars, etc.);
- [ ] Other: _______________.

To answer the following questions, simply select the desired option, ranging from 1 to 5, where:

1 – Strongly disagree	3 – Partially disagree	4 – Partially agree
2 – Partially disagree		5 – Strongly agree

**ATTITUDE**					
Reward from using functional foods					
09) Functional foods help improve my mood.	1	2	3	4	5
10) My performance improves when I consume functional foods.	1	2	3	4	5
11) Functional foods make it easier to adopt a healthy lifestyle.	1	2	3	4	5
12) I can prevent diseases by regularly consuming functional foods.	1	2	3	4	5
13) I enjoy the idea of taking care of my health by eating functional foods.	1	2	3	4	5
14) Functional foods can repair the damage caused by an unhealthy diet.	1	2	3	4	5
15) Even if the taste is unpleasant, I will consume food with functional properties.	1	2	3	4	5
16) I actively seek information about functional foods.	1	2	3	4	5
Need for Functional Foods					
17) Functional foods are completely unnecessary.	1	2	3	4	5
18) Functional foods are a complete scam.	1	2	3	4	5
19) The growing number of functional foods on the market is a bad trend for the future.	1	2	3	4	5
20) For a healthy person, using functional foods is not worth it.	1	2	3	4	5
21) It is great that technology allows the development of functional foods.	1	2	3	4	5
22) I only want to eat foods that have no medicinal/drug-like effects.	1	2	3	4	5

23) It is inappropriate to associate foods with health effects.	1	2	3	4	5
24) Functional foods are mostly consumed by people who do not need them.	1	2	3	4	5
25) Adding health benefits to unhealthy foods is pointless.	1	2	3	4	5
Trust in Functional Foods					
26) Functional foods promote my well-being.	1	2	3	4	5
27) The information available about the effects of functional foods is reliable.	1	2	3	4	5
28) The use of functional foods is completely safe.	1	2	3	4	5
29) The safety of functional foods has been thoroughly studied.	1	2	3	4	5
30) I believe that functional foods deliver the promised functions/characteristics.	1	2	3	4	5
31) Functional foods are the main products based on science.	1	2	3	4	5
Safety of Functional Foods					
32) When consumed in excess, functional foods can be harmful to health.	1	2	3	4	5
33) In some cases, functional foods can be harmful to healthy individuals.	1	2	3	4	5
34) The use of functional foods is completely safe.	1	2	3	4	5
35) The newly analyzed functionalities in foods present unforeseen risks.	1	2	3	4	5
36) Exaggerated information is provided about the health effects of functional foods.	1	2	3	4	5
**Reference Group**					
37) My family believes that eating functional foods is beneficial for health.	1	2	3	4	5
38) Nowadays, most health professionals approve of consuming functional foods.	1	2	3	4	5
39) My friends eat functional foods.	1	2	3	4	5

40) My family encourages me to consume functional foods.	1	2	3	4	5
41) Most people who care about me think that eating functional foods is good for my health.	1	2	3	4	5
42) Nutritionists recommend the consumption of functional foods.	1	2	3	4	5
**Beliefs**					
43) Functional foods are likely to have a beneficial impact on my health.	1	2	3	4	5
44) I try functional foods in an attempt to pursue a healthy lifestyle..	1	2	3	4	5
45) Consuming functional foods allows me to take care of my own health.	1	2	3	4	5
46) Including functional foods in my routine helps me meet my daily needs.	1	2	3	4	5
**Myths and Facts**					
47) Eating bananas at night is harmful.	1	2	3	4	5
48) Bananas cause constipation.	1	2	3	4	5
49) Mango with milk is harmful.	1	2	3	4	5
50) Lemon irritates the stomach.	1	2	3	4	5
51) Beetroot prevents anemia.	1	2	3	4	5
52) Gluten causes weight gain and is harmful.	1	2	3	4	5
53) Fiber intake is important for normal bowel function.	1	2	3	4	5
54) Consuming milk and dairy products causes inflammation in the body.	1	2	3	4	5
55) Carrots are good for vision and skin.	1	2	3	4	5
56) Eggs are associated with cardiovascular diseases and increased cholesterol.	1	2	3	4	5
57) Coconut oil aids in weight loss.	1	2	3	4	5
58) Garlic helps reduce blood pressure, lowers LDL (bad cholesterol), and increases HDL (good cholesterol).	1	2	3	4	5
59) Eggplant water aids in weight loss and reduces cholesterol.	1	2	3	4	5

60) Eating chicken is harmful due to the addition of hormones.	1	2	3	4	5
61) Gelatin improves skin elasticity due to collagen.	1	2	3	4	5
62)Drinking tea causes impotence.	1	2	3	4	5
